# Multiple Chemical Sensitivity: The Underrecognized Diagnosis but True Disease

**DOI:** 10.1192/j.eurpsy.2022.617

**Published:** 2022-09-01

**Authors:** V. Bellman, T. Zolnikov

**Affiliations:** 1 University of Missouri Kansas City, Department Of Psychiatry, Kansas City, MO, United States of America; 2 California Southern University, School Of Behavioral Sciences, Costa Mesa, CA, United States of America

**Keywords:** sensitivity, exposure, intolerance, toxicant

## Abstract

**Introduction:**

Multiple chemical sensitivity (MCS) is a chronic condition characterized by adverse health effects due to exposure to common chemicals which may lead to disability. The pervasive nature of stigma associated with MCS and similar conditions, including that which exists among providers, creates unbearable barriers to healthcare access.

**Objectives:**

The main objectives of this study are: (1) to describe the symptoms associated with MCS, (2) determine whether environmental exposure has an impact on psychological well-being of patients with MCS.

**Methods:**

The qualitative phenomenological study consisting of 42 individuals presenting with medically-unexplained symptoms was conducted using semi-structured interviews.

**Results:**

The symptoms experienced by participants with MCS are diverse, with common symptoms being migraine, paresthesias, seizure-like attacks, allergic reactions, respiratory symptoms (e.g., SOB, swollen throat), GI distress, muscle pain, chronic fatigue and persistent insomnia. These symptoms always develop in response to low level exposures to various toxicants, recur reproducibly and improve when toxic agents are removed. Finally, the adults with MCS are more likely to experience significant affective and PTSD-like reactions. The participants stated the stigmas and misconceptions against those with toxicant sensitivities affected their mental wellness.

**Conclusions:**

Multiple clinically significant behavioral and psychological symptoms are associated with MCS. Our data suggested that diagnostic overshadowing is pervasive in the healthcare system. This study also highlights the importance of psychological interventions and doctor–patient relationship in the management of MCS in various settings. Public education to increase knowledge around environmental illness is paramount.

**Disclosure:**

No significant relationships.

